# Bioengineered Interfaces for Peripheral Nerve Sensory Restoration

**DOI:** 10.1002/adma.74815

**Published:** 2026-08-27

**Authors:** Sydney Swedick, Salim El Hadwe, Kesi Liang, George G. Malliaras, Damiano G. Barone

**Affiliations:** ^1^ Department of Clinical Neurosciences Cambridge Biomedical Campus University of Cambridge Cambridge UK; ^2^ Electrical Engineering Division Department of Engineering University of Cambridge Cambridge UK; ^3^ Institute for Biomedical Innovation, Department of Engineering University of Cambridge Cambridge UK; ^4^ Department of Neurosurgery Houston Methodist Hospital Houston Texas USA; ^5^ Electrical and Computer Engineering Department Rice University Houston Texas USA; ^6^ Neuroengineering Initiative Rice University Houston Texas USA

**Keywords:** cognition, computer science, functional recovery, neuroscience, peripheral nerve injury, physical medicine and rehabilitation, rehabilitation, sensation, sensory system

## Abstract

Peripheral nerve injuries (PNIs) severely impair motor and sensory function, diminishing patient independence and quality of life. Despite decades of research, rehabilitation has prioritized motor recovery and residual function over the sensory restoration essential for embodiment, intuitive control, and natural movement. This lack of feedback drives poor prosthetic integration, high cognitive demand, and high abandonment rates. This review organizes the field along the translational pathway from injury to functional recovery: the biology of what is lost, strategies that restore the native substrate, the interfaces required when it cannot be rebuilt, and how these strategies are integrated and embodied. Approaches are compared by biological target, sensory function restored, invasiveness, clinical maturity, and limitation, emphasizing how interface properties such as modulus mismatch, charge injection capacity, and foreign body response govern long‐term stability and naturalness. Proprioception, the hardest and least measurable sensation to restore, is examined in depth, as are biomimetic, neuromorphic, biohybrid, and machine‐learning approaches toward adaptive, closed‐loop communication, weighed against their limits to adoption. The review closes with design principles and the challenges they face, from power and scalability to regulatory and ethical hurdles. The future lies in patient‐centered interfaces that let individuals not only move, but feel again.

## Graphical abstract

Half of amputees abandon their prosthetics for lack of feeling. This review charts the full path from peripheral nerve injury to restored sensation, through surgical, regenerative, noninvasive, and implanted approaches, and shows how injury type and interface material properties determine which strategy can deliver naturalistic feedback, and why proprioception remains the hardest problem to solve.

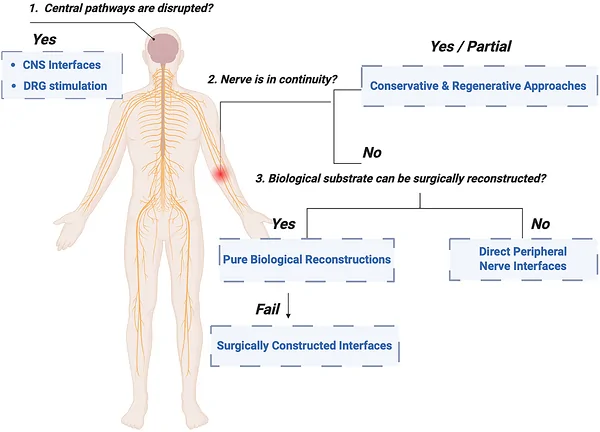

## 1 Introduction

Neurological disabilities affect 3.4 billion individuals worldwide, substantially affecting their health, finances, and social lives [1, 2]. Among those, limb loss is a major burden, with millions of people affected and prevalence expected to continue rising in the coming decades [3]. The main challenge in treating nervous system injuries is neurons’ limited regenerative capacity. After peripheral nerve injury (PNI), Wallerian degeneration breaks down the distal axon. Schwann cells form Bungner bands to guide axons and recruit macrophages to clear myelin debris, but this increases pro‐inflammatory cytokines and tissue necrosis [4]. Despite these repair mechanisms, axonal regeneration is slow and incomplete, as nerve growth factor (NGF) changes limit Schwann cells’ ability to support axon regeneration [5].

Efforts have largely focused on enhancing residual rather than reinstating lost functions, with emphasis on motor over sensory. The absence of sensory feedback—such as object manipulation, pressure modulation, and slip detection—has led to about 50% of amputees abandoning prosthetics due to poor embodiment, high cognitive load, discomfort, and residual pain [6]. Embodiment is the psychological integration of a prosthetic limb into one's body schema, such that the device is experienced as part of the body. Other factors influencing embodiment include alignment, appearance, early fitting, and patient education. Patient fitting within 3 months postamputation has been shown to significantly improve acceptance [7, 8]. Educating patients and customizing devices to their needs has also increased retention. To improve functionality, intuitiveness, and normalcy, there is a growing need to restore somatosensory feedback in prosthetics through afferent activation and proprioceptive cues [9]. The goal of much of this work is naturalistic sensation, or feedback that the brain interprets as a physiologically normal percept rather than as a learned, artificial signal. Surprisingly, despite their large diameter and low activation threshold, proprioceptive fibers are less studied than tactile. Recent technologies, however, show real promise to restore the sensory function that underlies coordinated movements, normal posture, and stable gait.

To chart that progress, this review follows the translational pathway from injury to functional recovery, evaluating each technology by biological target, sensory function, invasiveness, clinical maturity, and limitation.

## 2 Sensory Transduction and the Ascending Pathway

### 2.1 Tactile Feedback

Human sensory systems rely on a blend of tactile and proprioceptive information from afferent fibers to the brain, enabling precise control and object interaction [10]. The human hand, with 27° of freedom and tens of thousands of mechanoreceptive fibers, poses a challenge in scaling sensory encoding algorithms to match its complexity [11]. When touching objects, tactile receptors—Merkel disks, Meissner corpuscles, Ruffini endings, and Pacinian corpuscles—detect texture, slip, stretch, and vibration [12].

In glabrous skin, sensory fibers are further divided into rapidly adapting (RA), which respond to dynamic changes, and slowly adapting (SA), which respond to sustained stimuli [12]. These fibers are also classified by stimulus response. SA1 fibers, in Merkel disks, respond strongly at initial contact and decrease over time, sensitive to edge contours [12]. SA2 fibers, in Ruffini endings, respond steadily during contact and to skin stretch, potentially signaling joint position [12]. Similarly, in RA nerve fibers, RA1 fibers, in Meissner corpuscles, respond to small, changing indentations, while RA2 fibers, in Pacinian corpuscles, detect rapid onsets/offsets and high‐frequency vibrations. RA1 and SA1 offer high tactile resolution for localizing contact and surface detail, while RA2 and SA2 have larger receptive fields with less detail [12].

The final category of sensory fibers is C‐tactile (CT) fibers, unmyelinated low‐threshold mechanoreceptors, found in hairy skin, the face, and glabrous skin of the hands that respond to slow, light touch, playing a role in affective touch [13]. The various types of mechanoreceptors and proprioceptors are illustrated in Figure 1.

**FIGURE 1:**
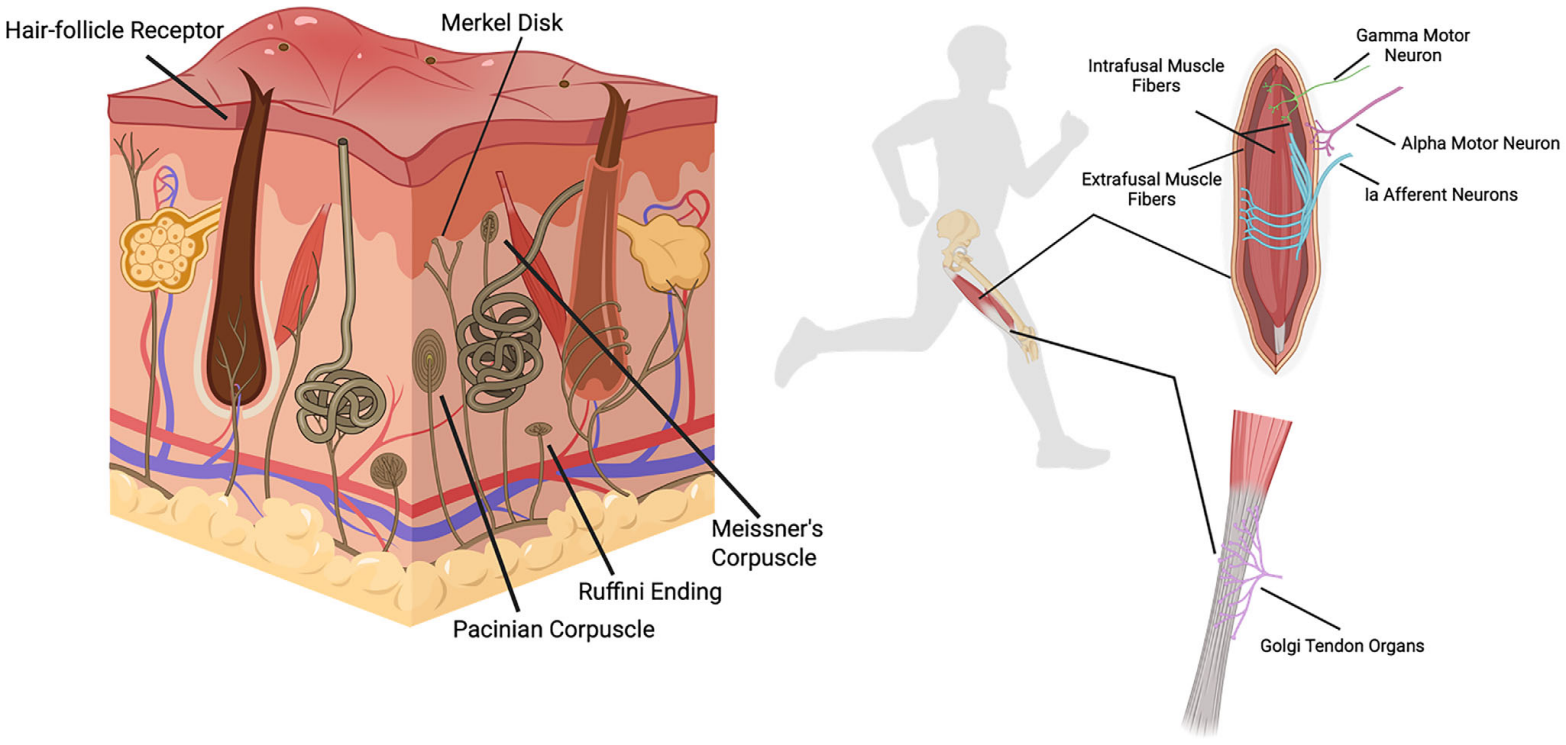
Sensory receptors in skin and musculoskeletal system. The image shows mechanoreceptors in skin (left) and proprioceptors in muscles and tendons (right). Cutaneous receptors include Merkel disks and Meissner's corpuscles (light touch), Ruffini endings (skin stretch), Pacinian corpuscles (vibration and pressure), and hair‐follicle receptors. Proprioceptors include muscle spindles (intrafusal fibers, gamma motor neurons, Ia afferents) for muscle stretch sensing, and Golgi tendon organs (GTO) at the muscle‐tendon junction, detecting tension. These inputs contribute to touch perception and proprioception.

### 2.2 Proprioceptive Feedback

Proprioception is the sense of body position and movement in relation to the external world, essential for balance, posture, gait, and object manipulation [14]. The key proprioceptive receptors include muscle spindles within skeletal muscles, which convey posture and movement, and GTOs located at the muscle‐tendon junction, which signal tendon tension and muscle load [15].

### 2.3 From Stimuli to the Somatosensory Cortex

Sensory information is encoded and transmitted via sensory nerves to the brain's somatosensory cortex (S1). Peripheral nerves, composed of cable‐like fibers varying in number and size, are covered in connective endoneurium tissue, organized into somatotopic fascicles brought together by the perineurium, and bundled into loosely connecting groups of fascicles by the epineurium. Motor (efferent) and sensory (afferent) nerves are bundled separately and innervate distinct areas of muscle and skin. Sensory signals, as shown in Figure 2, pass along the afferent fibers to the pseudounipolar dorsal root ganglion (DRG) in the spinal cord's intervertebral foramina. The information then enters the spinal cord, ascends to the cuneate nucleus (CN) in the brainstem, and onto the ventroposterior nucleus (VPN) of the thalamus [15].

**FIGURE 2:**
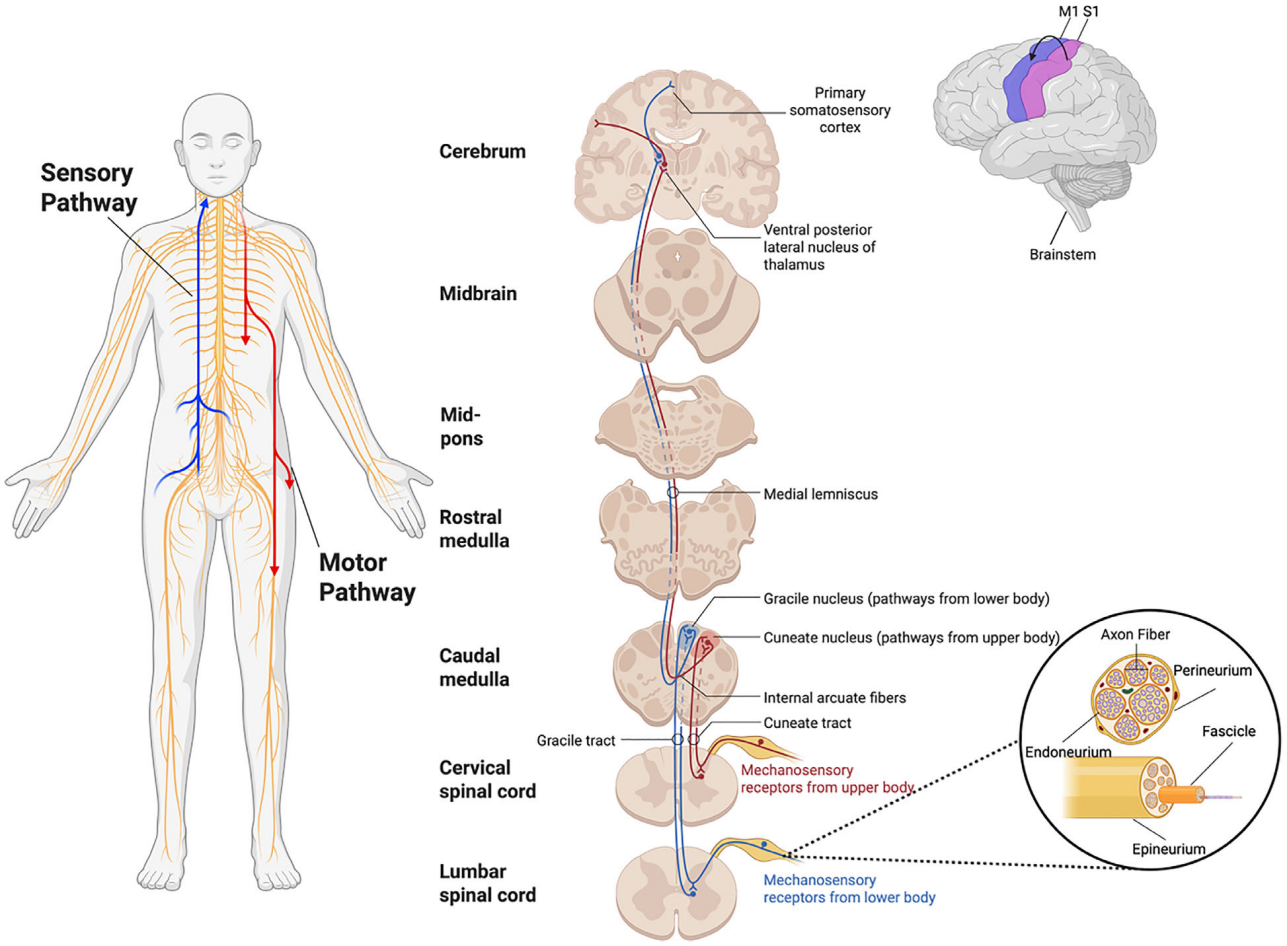
Overview of the ascending sensory pathway. Depicts from mechanoreceptors to the primary somatosensory cortex (S1), then to the motor cortex (M1) to influence movement. Inset shows peripheral nerve structure.

S1, located in the postcentral gyrus, includes Brodmann's areas 3a, 3b, 1, and 2. Area 3a receives proprioception and 3b and 1 receive tactile information, while area 2 integrates proprioceptive and tactile information [16, 17, 18]. Representations of body parts within S1 are not distributed evenly and instead depend on receptor density, giving fingertips and hands larger representations. Moreover, low‐pressure stimuli cause localized S1 activation, while high‐pressure stimuli cause more widespread activation.

Once in S1, information is transmitted to the motor cortex (M1), influencing motor function and real‐time corrections. Severe motor learning deficits occur in the absence of S1, indicating the significance of S1 in modulating motor function [19]. Importantly, nerve injury destroys sensory end‐organs but not the pathways connecting them to the brain, allowing stimulation or regeneration to recreate sensory perception.

## 3 Peripheral Nerve Therapies for Sensory Restoration

Damage to the sensory pathway can be addressed at three levels—the cortex, the spinal cord, or the peripheral nerve—each with different trade‐offs in selectivity, naturalness, and risk. Of these, peripheral nerve repair offers the most favorable balance for treating sensory deficits. Cortical interfaces bypass natural pathways, while peripheral nerve devices restore full somatosensory pathways [20, 21]. Cortical interfaces also pose surgical risks, size constraints, paresthesia from unnatural fiber activation, and proximity to vital brain regions, increasing the risk for hemorrhage, stroke, and infection [20]. Due to similar diameters and stimulation thresholds among mechanoreceptors in the brain, it is difficult to selectively activate neuronal populations without activating unintended areas, causing unknown consequences [22]. Spinal cord interfaces, while less invasive than cortical approaches, suffer from low selectivity and poor spatial resolution, and like cortical stimulation they act upstream of the natural sensory encoding [23, 24]. Instead, targeted peripheral nerve therapies emulate natural signaling from mechanoreceptors, thermoreceptors, and nociceptors. While they may not be natural at the axonal level, they preserve thalamic relays and projections to S1, which the brain classifies against previous sensory experience to promote natural perception [25].

The strategies for sensory restoration can be broadly organized into three categories, which guide the comparisons throughout this review. First, a therapy is homologous when it restores sensation through the original sensory modality (e.g., electrical stimulation of sensory fibers) and nonhomologous, or a sensory substitution, when it uses a different modality (e.g., vibrotactile cues for skin stretch) [26]. Second, feedback is somatotopic when the sensation is felt in the anatomically natural region and nonsomatotopic when it is referred elsewhere [26]. Third, approaches differ in invasiveness, which trades selectivity and naturalness against surgical risk. Peripheral nerve interfaces are attractive because they are often both somatotopic and homologous, but this comes at the cost of invasiveness.

No one solution fits all. However, the most effective approach matches the original sensation's modality, location, and signal properties to the injury. Restoring existing pathways gives the most physiologically relevant feedback but is not always possible. The optimal intervention depends on the nature and location of the injury, the residual neural and muscular architecture still prevalent, and the functional goals of the patient. Broadly, injuries that sever the pathway to the brain, as in complete spinal cord injury, leave central nervous system interfaces as the only route. For PNIs, those where the nerve remains in continuity favor conservative and regenerative approaches, while a severed nerve is either surgically reconstructed through repair, grafting, or targeted reinnervation, or, where reconstruction is not possible, engaged directly through implanted electronic interfaces. Table 1 compares all major approaches discussed in this review along the defined categories, while Figure 3 maps injury type and severity onto the most suitable class of interface. The following sections apply this framework, moving through the major approaches in turn.

**TABLE 1:** Comparative landscape of approaches for peripheral nerve sensory restoration.

Categories	Approaches	Biological Targets	Sensory Function Restored	Homologous	Somatotopic	Invasiveness	Clinical Maturity	Principal Limitation	Refs.
Pure biological reconstructions	Hand/limb transplants	Whole limb anatomy	Comprehensive tactile & proprioception	Yes	Yes	Highly invasive	Clinical	Lifelong immunosuppression; high risk of rejection/infection; profound psychiatric/psychosocial burden	[42, 43]
	Sensate flaps (ALT/Foot)	Subcutaneous tissue / mechanoreceptors	Tactile (protective sensation)	Yes	Yes (local)	Surgical	Clinical	Donor site morbidity; complex microsurgery	[44, 45]
	Glabrous skin Grafts	Skin mechanoreceptors	High‐resolution tactile	Yes	Yes (local)	Surgical	Clinical	Limited donor tissue availability	[28, 46]
	Distal nerve transfers	Sensory nerve/mechanoreceptors	Local tactile/sensory feedback	Yes	Yes	Surgical	Clinical	Requires sufficient donor nerve length; limited donor availability	[32]
Surgically constructed interfaces	TSR	Cutaneous sensory end‐organs	Touch, vibration, temperature, pain	Yes	Yes (referred)	Surgical	Clinical	Sacrifices native chest sensation; low‐resolution/variable sensory maps	[27, 28]
	TMR	Denervated target muscles	N/A (Motor control/Pain reduction)	No	No	Surgical	Clinical	No functional tactile or proprioceptive restoration	[29, 30, 31]
	AMI	Tendon‐connected muscle pairs	Proprioception	Yes	Yes	Highly invasive	Early human	Requires specific reconstructive amputation; potential muscle atrophy	[34, 35]
	CMI	Skin flaps/mechanoreceptors	Graded touch, vibration	Yes	Yes	Surgical	Research	Requires available skin flap tissue; risk of scarring/neuroma	[36]
	RPNI (and variants)	Denervated muscle grafts / dermal skin grafts	Pain reduction (RPNI); Tactile (cRPNI / DS‐RPNI)	Partial / Variant‐dependent	Partial / Variant‐dependent	Surgical	Clinical (RPNI)/research (cRPNI/DS‐RPNI)	Requires autologous graft tissue; no proprioception	[37, 38, 39]
	Scaffold & sieve electrodes (TEENI)	Proximal nerve stump/regenerating axons	Bidirectional high‐resolution tactile & proprioceptive feedback	Yes	Yes	Highly invasive	Research	Slow axonal regeneration; high risk of target misrouting	[40, 41]
Noninvasive interfaces	Electrotactile	Surface tactile afferents	Touch, pressure (paresthesia)	No	No	Noninvasive	Clinical	Skin irritation/discomfort; frequent recalibration required	[26, 58, 59, 60]
	Vibrotactile	Skin mechanoreceptors (Pacinian corpuscles)	Simple tactile/vibration cues	No	No	Noninvasive	Clinical	Sensory adaptation (numbness); low information bandwidth	[26]
	Mechanotactile	Skin mechanoreceptors	Graded force, skin stretch	Yes	No	Noninvasive	Research/early human	Actuators are bulky, heavy, and power‐intensive	[61, 62, 63]
	TENS	Sensory nerves & superficial tactile receptors	Tactile feedback, object slippage, pressure shifts, & phantom pain reduction	Yes	Yes	Noninvasive	Clinical	Poor selectivity compared to invasive methods; difficulty aligning perceived sensation	[33, 57, 64, 65, 66, 67, 68]
	Artificial fingertips	Native tactile receptors	Object manipulation, grasp control, slip detection	Yes	Yes	Noninvasive	Research	High variability in skin properties (thickness, stiffness, age) limits accuracy and usability	[54]
	Thermal wearables (eg., MiniTouch)	Temperature‐sensitive A‐delta fibers (Thermoreceptors)	Natural thermal sensation	Yes	Yes	Noninvasive	Research	Difficulty selectively stimulating small A‐delta fibers without unwanted activation of pain fibers	[54, 80, 81, 153]
	Ultrasound stimulation	Deep muscles and peripheral nerves	Spatial control and tactile feedback	No	Yes	Noninvasive	Research	Issues with device size, interfacing, and calibration	[64]
	TENGs & multimodal haptic gloves	Skin mechanoreceptors & superficial nerves	Tactile feedback (hardness, roughness)	Partial / Combined	No / Referred	Noninvasive	Research	Requires miniaturization and energy efficiency; risk of unwanted muscle activation	[65, 66]
	AR	Visual and auditory pathways	Proprioception and grip feedback	No	No	Noninvasive	Research	Interferes with normal vision and hearing, severely limiting real‐world use	[65]
Invasive and implantable interfaces	Extraneural electrodes (e.g., Cuffs, FINE)	Epineurium	General tactile feedback, grasp modulation	Yes	Yes	Minimally invasive	Research/clinical	Fibrous encapsulation; high current requirements risk off‐target motor activation and paresthesia	[70, 81, 146, 158]
	Intraneural electrodes (e.g., LIFE, TIME, USEA)	Intrafascicular axons	High‐resolution, discrete tactile & proprioceptive feedback	Yes	Yes	Highly invasive	Research/early human	Severe FBR; mechanical stiffness mismatch causes chronic tissue damage and progressive signal degradation	[82, 83, 84, 85, 86, 87, 127, 147]
	DRG Stimulation	Dorsal root ganglion	Tactile & proprioceptive feedback	Yes	Yes	Highly invasive	Research/clinical	Extreme surgical complexity; risks of CSF leaks, infection, and lead migration	[14, 68, 69]

*Surgical* = Standard microsurgery. *Highly Invasive* = Extreme anatomical redesign or penetrating sensitive neural tissue. *Clinical* = Standard of care; available. *Early Human* = First‐in‐human success, but highly experimental and limited to a few patients.

**FIGURE 3:**
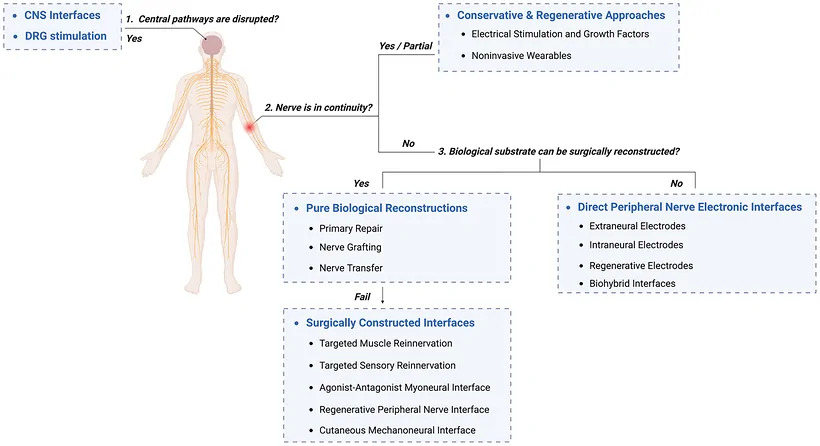
Decision framework mapping injury type and severity to interface class. A sequential decision tree routes an injury by whether central pathways are intact, whether the nerve is in continuity, and whether the substrate is surgically reconstructable, toward CNS interfaces, conservative and regenerative approaches, biological reconstructions, surgically reconstructed interfaces, or direct electronic interfaces.

## 4 Restoring the Native Substrate: Surgical Innovations and Regenerative Medicine in Peripheral Nerve Injury Treatment

Restoring the native substrate is the first option. Since peripheral nerves have a limited regenerative capacity, recent advances in surgical techniques and regenerative medicine aim to restore anatomy, providing a conducive environment for regeneration.

### 4.1 Nerve Transfers for Sensory Restoration

Nerve transfer, a common approach for restoring function after amputation or proximal nerve injury, reroutes intact donor nerves to denervated targets. Two of the most common are targeted sensory reinnervation (TSR), a leading surgical approach for restoring sensation, and targeted muscle reinnervation (TMR), which provides the motor control required for closed‐loop prosthetic control. TSR reroutes sensory end‐organs to chest skin, enabling perceived touch on a missing limb through robotic actuators that mimic fingertip sensation [27]. However, creating these end‐organs comes at a cost, most often chest sensation is sacrificed for hand sensation, so native and reinnervated perceptions blend [28]. Vibrotactile feedback, mechanical pressure, and transcutaneous stimulation further enhance the correlation between the location of the perceived sensation and pressure on the prosthetic hand [28]. TSR requires only one surgery, avoids implants, and provides anatomical, modality‐matched feedback with long‐term stability, restoring touch, vibration, temperature, force, and pain in the missing limb nearly to normal levels with subsequent embodiment [27]. TMR redirects motor nerves from the amputated limb to chest muscles, promoting intuitive control of myoelectric devices by detecting muscle contractions, reducing neuroma formation [29]. Initially for shoulder disarticulations and above‐elbow amputations, TMR is now used for transradial, transfemoral, and transtibial amputations [30, 31]. Integrating TMR and TSR with neural machine interfaces (NMIs) allows for intuitive closed‐loop prosthesis control [30]. When the individual tries to move their missing limb, reinnervated muscle contracts, and the muscle activity signal makes directed decision‐based movements.

Similarly, distal nerve transfers work through connecting donor nerves to nearby target muscles for faster recovery with fewer complications. For example, transferring the abductor digiti minimi (ADM), a branch of the ulnar nerve, helps restore median nerve sensation and thumb function more effectively than tendon transfers, though with longer recovery times [32]. However, sensory nerve transfers are still in early stages of research compared to motor nerve transfers.

These techniques leverage neuroplasticity, remapping sensory cortical areas, and promoting cortical activation like that of healthy individuals [33]. Still, TSR is limited by its reliance on available residual skin, unpredictable reinnervation that limits spatial resolution, its inability to restore proprioception, and its recalibration demands—limitations that motivate interfaces capable of engaging the peripheral nerve more directly and selectively (Table 1).

### 4.2 Advanced Prosthetic Surgical Interfaces

Emerging prosthetic surgical techniques, such as agonist‐antagonist myoneural interfaces (AMI), cutaneous mechanoneural interfaces (CMIs), and regenerative peripheral nerve interfaces (RPNIs), surgically reconfigure residual muscle and nerve into dedicated biological channels for richer prosthetic control and feedback, including the proprioception that standard nerve transfers cannot provide, with their procedures depicted in Figure 4.

**FIGURE 4:**
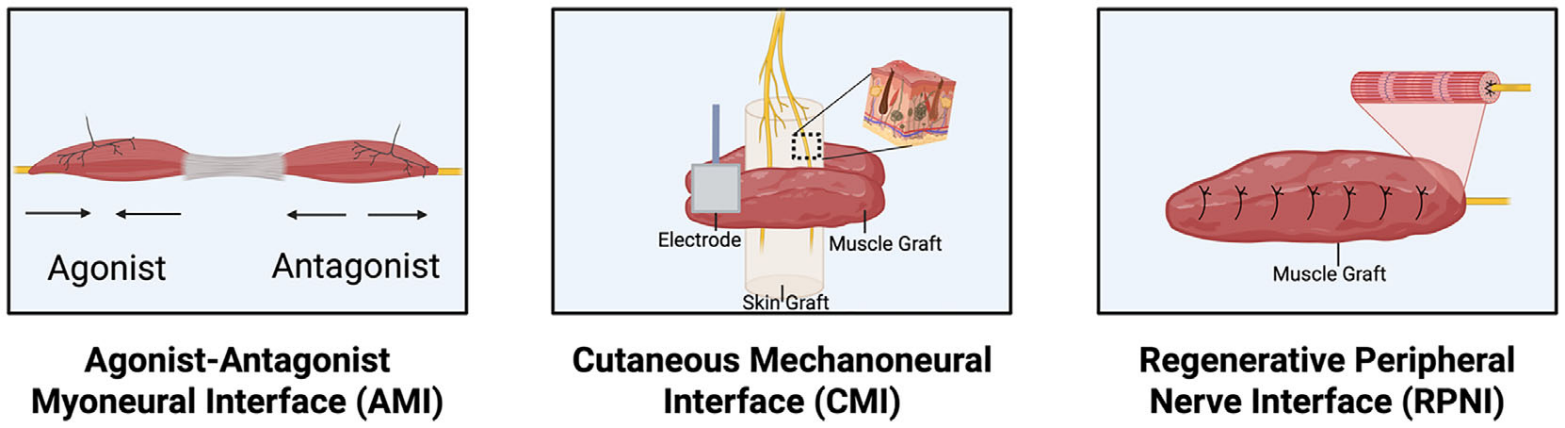
Neuroprosthetic interfaces for motor and sensory integration. Three biologically inspired interfaces are shown: the Agonist‐Antagonist Myoneural Interface(AMI) enables bidirectional motor control via dynamic muscle pairings, the Cutaneous Mechanoneural Interface (CMI) uses a skin graft innervated by peripheral nerves and anchored by a muscle graft to restore sensation and cutaneous feedback, and the Regenerative Peripheral Nerve Interface (RPNI) employs a muscle graft innervated by a transected nerve to facilitate motor signal transduction and control. Each leverages biological substrates to support reinnervation, signal transduction, and potential prosthetic integration, advancing toward closed‐loop motor and sensory restoration.

AMI uses tendon‐connected muscle pairs to restore proprioception. Electrical stimulation preserves muscles contractility and prevents atrophy in amputees [34]. When one muscle contracts, the opposing muscle stretches, activating muscle spindles and GTOs to signal joint position, speed, and force for control. AMIs have been successfully implemented, notably in the Ewing amputation, where a transtibial procedure utilized two native AMIs for greater proprioception and prosthetic control [35]. Unlike TMR or RPNIs, AMIs maintain the agonist‐antagonist muscle relationship, allowing for intact pathways of muscle spindles and GTOs.

CMIs use muscle‐actuated skin flaps and native mechanoreceptors to convert mechanical deformation from prosthetic sensors into afferent signals [36]. With functional electrical stimulation (FES), CMIs produced at least four levels of graded touch and eight vibrations comparable to healthy skin [36]. Challenges include scar tissue formation and neuromas, and limited stump skin has driven interest in grafting transected tactile nerves, as in dermal sensory RPNIS (DS‐RPNIs) [37].

RPNIs more broadly use denervated muscle grafts coapted to distal amputated nerves, restoring neuromuscular junctions and myoelectric control [38]. Variants include composite RPNIs (CRPNIs) and DS‐RPNIs, which provide touch and vibration but not proprioception and require motor integration [39]. Tissue‐engineered electronic nerve devices (TEENIs) embed flexible electrodes in hydrogel scaffolds for nerve regeneration after transection [40], and sieve electrodes use perforated holes for nerve regrowth [41]. These advancements enhance functional restoration and support seamless integration with native tissue.

### 4.3 Surgical Grafts and Flaps for Restoration

When tissue loss extends beyond the nerve to the skin and structures that carry sensation, reconstruction aims to restore the sensate tissue itself through anterolateral thigh (ALT) flaps, sensate foot flaps, glabrous skin grafts, and transplantation. The first hand transplant failed due to inadequate immunosuppression. Since then, microsurgery and immunosuppressive therapy advancements have made reconstructions, transplants, flaps, and grafts possible [42]. Today, many transplanted hands survive long term, with restored sensory and motor feedback. However, rejection, metabolic complications, infections, and the financial burden of lifelong immunosuppression are still concerns with these approaches. Careful patient selection, psychiatric evaluations, and psychosocial aspects must be considered before suggesting these treatments as feasible options [43].

Vascularized flaps aid in hand and multi‐digit reconstructions through immediate mobilization, minimizing scarring and increasing functional and cosmetic outcomes [44]. A study with 39 patients showed chimeric ALT and multilobed dorsalis pedis artery (DPA) flaps for reconstructing hands, resulting in functional and protective sensation restoration [44]. ALT flaps help to minimize donor site morbidity and reduce the need for multiple flap transfers, while multilobed DPA flaps are thin and pliable, providing texture like the palm for functional restoration [44].

Sensate foot flaps for digital reconstruction minimize morbidity and provide a better cosmetic option [45]. Glabrous skin within the foot flaps is highly innervated with mechanoreceptors compared to nonglabrous and provides the necessary texture, weight, and pliability for reconstruction [45]. Glabrous skin grafts have shown that when they reinnervate nonglabrous skin, the nerves regain sensitivity to the same degree as native skin [28]. Additionally, when used instead of nonglabrous skin, the reinnervated nerves do not compete with the native afferent fibers [46].

While these options do not provide yet restoration to the level of TMR and TSR, they do showcase the remarkable advancements of reconstructive surgery and the possibilities of the future. As surgical techniques continue to improve, their combination with bioelectronic interfaces will pave the way toward closed‐loop bidirectional restoration.

### 4.4 Regenerative Medicine for Nerve Restoration

When the nerve itself is salvageable, the goal shifts from reconstructing lost tissue to supporting the nerve's own regeneration and improving how it integrates with implanted devices. Regenerative biology aims to bridge the gap between neural tissue and electrodes, reducing foreign body response (FBR), an immune defense against foreign implanted materials [47]. One approach involves penetrating electrodes coated with neural growth factors that promote migration of the native neural tissue to the electrode interface, leading to direct, high‐resolution signaling [48]. On the cellular side, advances using mesenchymal stem cells (MSCs), bone marrow stem cells, and Schwann cells, have enhanced peripheral nerve regeneration [49].

Electrical stimulation itself is regenerative. It promotes peripheral nerve regeneration by driving calcium signaling and upregulating key neurotrophic factors like brain‐derived neurotrophic factor (BDNF) and its receptor tropomyosin receptor kinase B (TrkB). BDNF overexpression prevents degradation of cyclic adenosine monophosphate (cAMP), which boots regeneration genes like Ta1 tubulin and p‐43 [50]. Stimulation can also activate Schwann cells to secrete glutamate, rising intracellular calcium, and triggering the p38 MAPK pathway to promote neurite outgrowth [51, 52]. Just one hour of stimulation can enhance axonal regeneration and is more effective than surgery alone [53, 54]. Success depends on injury severity and timing, requiring careful patient selection. A constraint on all substrate‐level approaches is time, axons regrow at only 1 mm/day, so for limb‐level injuries spanning more than 30 cm, regeneration can take months to years. During that window axons frequently misroute to incorrect targets, denervated muscle atrophies, the neuromuscular junction degenerates, and cortical reorganization proceeds, so even successful peripheral reinnervation can mismatch what the cortex expects and yield poor functional integration [55].

## 5 Peripheral Nerve Interfaces for Sensory Feedback

When the native substrate cannot be fully restored, sensory feedback can instead be delivered directly through peripheral nerve interfaces. These fall broadly into noninvasive and invasive approaches, which trade selectivity and naturalness against surgical risk. Noninvasive interfaces are more affordable and don't require surgery but sacrifice nerve specificity and can elicit fewer natural sensations [56]. Invasive interfaces, by contrast, target nerves directly with smaller charge densities, reducing tissue damage, and offering more natural sensation, but at the cost of surgical risk [56].

### 5.1 Noninvasive Interfaces

Noninvasive interfaces deliver sensory feedback without surgery, improving function and user experience after PNIs, especially for restoring upper‐limb sensation to detect contact timing, pressure, and slippage [10, 57]. They span a growing range of modalities, from haptic feedback applied to the skin, to cross‐modal substitution through other senses, to emerging techniques that aim to recreate naturalistic sensation.

#### 5.1.1 Haptic Feedback

Haptic feedback aids grip and object manipulation in prosthetic users, categorized into electrotactile, mechanotactile, and vibrotactile feedback, as seen in Figure 5. Electrotactile and vibrotactile are sensory substitutions, while mechanotactile is modality‐matched but location‐mismatched.

**FIGURE 5:**
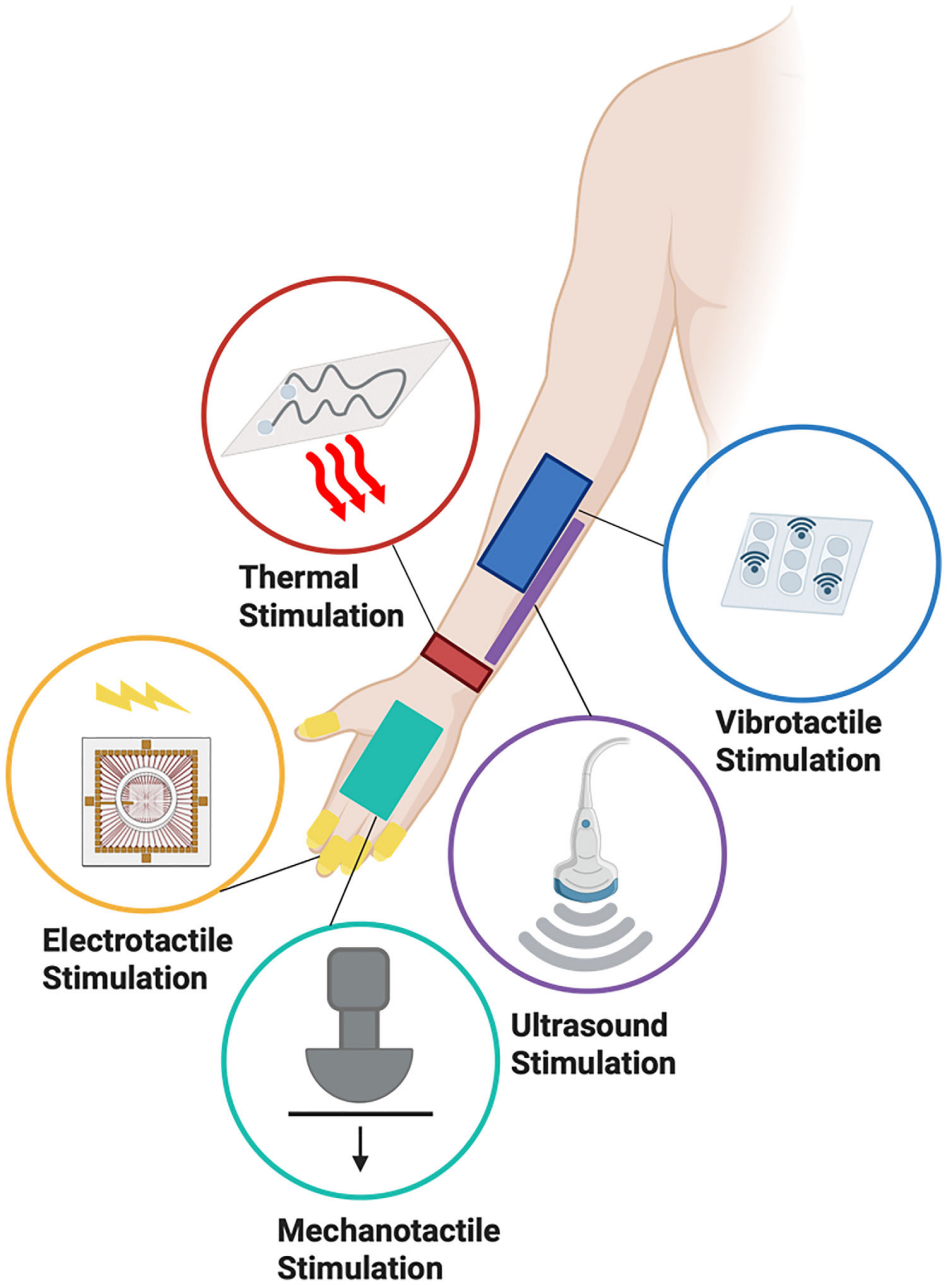
Types of haptic feedback. Illustrated on the forearm: thermal (red), vibrotactile (blue), electrotactile (yellow), ultrasound (purple), and mechanotactile (green), each targeting sensory receptors and nerves via different modalities.

Electrotactile feedback is compact, low‐power, high‐resolution, and customizable, using mild electrical pulses to activate superficial tactile afferents without activating deeper motor neurons, causing muscle contractions. Arrays deliver touch, pressure, and vibration by adjusting intensity, pulse width, and frequency [26, 58]. Sensation location and spread depend on stimulation pattern resolution and electrode configuration [59]. Electrotactile feedback can be somatotopically matched for better results using hand maps on the stump, though many amputees lack or have incomplete maps [60]. Challenges with electrical stimulation include uncomfortable, unnatural sensations from not properly adjusted parameters and sweat‐related impedance shifts requiring constant recalibration [26]. Better parameter modulation is needed for effective sensory encoding and user comfort. Still, electrotactile arrays are light and power‐efficient alternatives to skin‐stretch mechanotactile or vibrotactile systems. Mechanotactile feedback applies normal or shear forces to deform the skin, improving grasp and object manipulation [61]. Vibrotactile feedback, a subtype, uses mechanical devices to vibrate the skin near the amputation [26]. Users learn to associate vibration patterns based on amplitude and frequency with tactile events, though irritation, pain, and desensitization to the feedback come with prolonged use. Vibrotactile feedback is best used occasionally for short periods for simple and more natural feedback than electrical stimulation. Mechanotactile is superior to vibrotactile feedback due to its modality‐matched nature [62], and skin‐stretch devices are favored for conveying proprioceptive position, direction, and force [63], though devices such as the stretch‐pro remain heavy and energy‐intensive, limiting adoption [63].

Beyond mechanical actuation, transcutaneous electrical nerve stimulation (TENS) offers a promising noninvasive route to somatotopic feedback, stimulating sensory nerves or tactile receptors through surface electrodes, eliciting richer sensations than mechanical touch [64]. The variability in sensation intensity and area elicited by frequency or pulse‐width modulation, which also varies with current, electrode type, skin thickness, and receptor density, allows fine‐tuned feedback, essential for tasks requiring tactile precision [64]. When eliciting tactile feedback in specific locations of the residual limb corresponding to areas on the missing limb, TENS creates somatotopically evoked tactile sensations, leveraging neuroplasticity of the body representation from nerve reinnervation [33, 64]. By exploiting this phenomenon, TENS can also be used for phantom pain management and the embodiment of prosthetic devices [65]. Difficulties can arise with aligning the perceived sensation with the intended location through TENS. However, strategies using somatotopic feedback utilizing phantom hand maps still create the most intuitive feedback, and TSR can generate maps in those without them [66].

TENS has advanced with apparent moving sensation (AMS), which mimics skin motion by modulating stimulus timing and intensity, conveying object slippage and pressure shifts during gait [57]. AMS relies on pulse amplitude, width, and inter‐stimulus delay modulation, while multiparametric TENS enhances naturalistic somatosensation [57, 67]. Future work for TENS should include closed‐loop systems for tactile and slippage feedback and adaptive parameters based on psychophysiological signals like EEGs [57, 68, 69]. Though less selective than invasive methods, TENS offers a wide range of usability, including lower limb amputees, with long‐term stability [70].

#### 5.1.2 Multimodal and Cross‐Modal Sensory Modalities

Multimodal sensory integration combines multiple modalities to replicate complex sensations, but can overwhelm users with too much information at once, lowering performance. Sensory substitution techniques, like sound‐to‐touch conversion, also show promise through spatiotemporal vibrotactile skin patterns to convey the environment. However, they still have challenges of vibrotactile feedback, including low resolution and bulkiness [71].

These issues have driven interest in cross‐modal feedback, where one sensory modality influences another, specifically audio‐visual (e.g., lip reading) and visual‐tactile (e.g., feeling touched through visual instead of physical sensation). Visual feedback helps direct movements but lacks tactile feedback for delicate tasks. Auditory feedback is fast, low‐power, and provides information on force and proprioception by modifying pitch or volume of the signal [72]. Nevertheless, auditory and visual substitutions can interfere with normal sensory processing, limiting usability.

#### 5.1.3 Emerging Noninvasive Techniques and Current Challenges

The newest noninvasive approaches aim less at delivering a single substituted cue and more at recreating the full richness of natural touch, spanning artificial fingertips, ultrasound stimulation, augmented reality (AR), and biomimetic systems.

Artificial fingertips simulate tactile feedback for object manipulation, grasp control, and slip detection through high‐resolution sensors, but they still lack everyday usability [73]. Difficulties arise in recreating fingertip sensation due to varying skin properties like thickness, stiffness, and individual factors like age and gender.

Beyond tactile feedback, thermal sensation is another critical area of sensory restoration. New noninvasive wearables recreate physiologically normal thermal sensations enhancing embodiment [74]. Some integrate mechanical and thermal cues to simulate different temperatures during touch [75]. The MiniTouch system shows a promising future distinguishing thermal stimuli in commercial prosthetics [76, 77]. Accuracy can vary due to the difficulty of stimulating temperature‐sensitive A‐delta fibers from their small size and size overlap with unwanted activation of pain fibers.

Other emerging technologies, like ultrasound stimulation, AR feedback, and triboelectric nanogenerators (TENGs), are also promising. Ultrasound stimulation can penetrate deep muscles for spatial control but faces issues with size, interfacing, and calibration [78]. AR feedback has been used to improve proprioception and grip feedback [79], though results vary. AR also interferes with normal vision and hearing, limiting its real‐world use, but remains useful for training and device testing. For example, TENG systems have been introduced into the virtual world, converting mechanical energy into precise, painless electrical stimulation for tactile feedback offering promise for self‐powered, flexible wearables [79]. Similarly, haptic gloves combining vibrotactile, thermoelectric, and electrotactile systems recreate complex sensations like hardness and roughness, enabling intuitive robotic hand control in the virtual world [80].

Across these noninvasive modalities, readiness varies widely for practicality in daily use. Electrotactile and vibrotactile systems are the most mature and commercially available but the least natural. Mechanotactile skin‐stretch and the MiniTouch thermal system are the strongest candidates for near‐term naturalistic feedback because they are modality‐matched, while ultrasound, AR, and TENG remain investigational. However, noninvasive interfaces still share many challenges, including discomfort and poor selectivity, with habituation from high charge densities near electrodes linked to tissue damage [81]. Channel‐hopping interleaved pulse scheduling (CHIPS) distributes pulses across electrodes, lowering charge per pulse while preserving total charge to create stronger, more selective, more comfortable sensations [81]. Even so, miniaturization, energy efficiency, user‐specific customization, and recreating homologous proprioceptive feedback without unintended muscle activation remain the principal barriers to widespread noninvasive adoption.

### 5.2 Invasive and Implantable Interfaces

Invasive stimulation targets nerves instead of muscles, using smaller charge densities, reducing tissue damage and power consumption, while offering more natural sensation, but with risks of infection and rejection [56]. DRG stimulation exploits the separation of sensory and motor neurons to selectively recruit somatosensory afferents via epineural electrodes with limited motor activation, recruiting proprioceptive afferents in felines [14, 23, 24].

Implantable electrodes evoke somatosensory percepts directly and are classified by invasiveness and selectivity seen in Figure 6. Extraneural electrodes like helical and flat interface nerve electrodes (FINE) encapsulate the nerve without penetrating it, minimizing tissue disturbance while maintaining long‐term stability [82, 83]. The compliant and flexible interface for nerve electrodes (C‐FINE), made of pliable silicone, balances stiffness and flexibility for nerve movement [84]. Each design has trade‐offs, as circular designs reduce tissue contact through a smaller surface area, minimizing damage, while flattened designs increase surface area for stimulation without penetration [82]. Though less invasive, extraneural electrodes often need higher stimulation currents, risking unwanted neuron activation and paresthesias [85]. These effects can be minimized by adjusting stimulation timing [25]. Intraneural interfaces insert electrodes into the nerve to target specific fascicles, enhancing electrode‐axon contact. However, effectiveness is limited by the random placement of Nodes of Ranvier, short unmyelinated sections along the axon limiting their ability to conduct a signal, as well as axon size variation, and spatial constraints. Specific designs penetrate through the epineurium, like the slowly penetrating interfascicular nerve electrode and the groove electrode [86, 87], while others also penetrate through the perineurium, such as the intrafascicular electrode (LIFE), transverse intrafascicular multichannel electrode (TIME), and Utah slanted electrode array (USEA) [88, 89, 90]. LIFE electrodes, made of polyimide or parylene, have variants like FAST‐LIFEs (LIFE and fascicular cuff electrode) and tf‐LIFEs (parylene‐C, platinum, and iridium oxide), which improve stability, biocompatibility, and charge capacity [91, 92, 93]. Despite comparable function to extraneural electrodes, intraneural interfaces have issues with high stimulation thresholds and inflammatory responses limiting stability [92].

**FIGURE 6:**
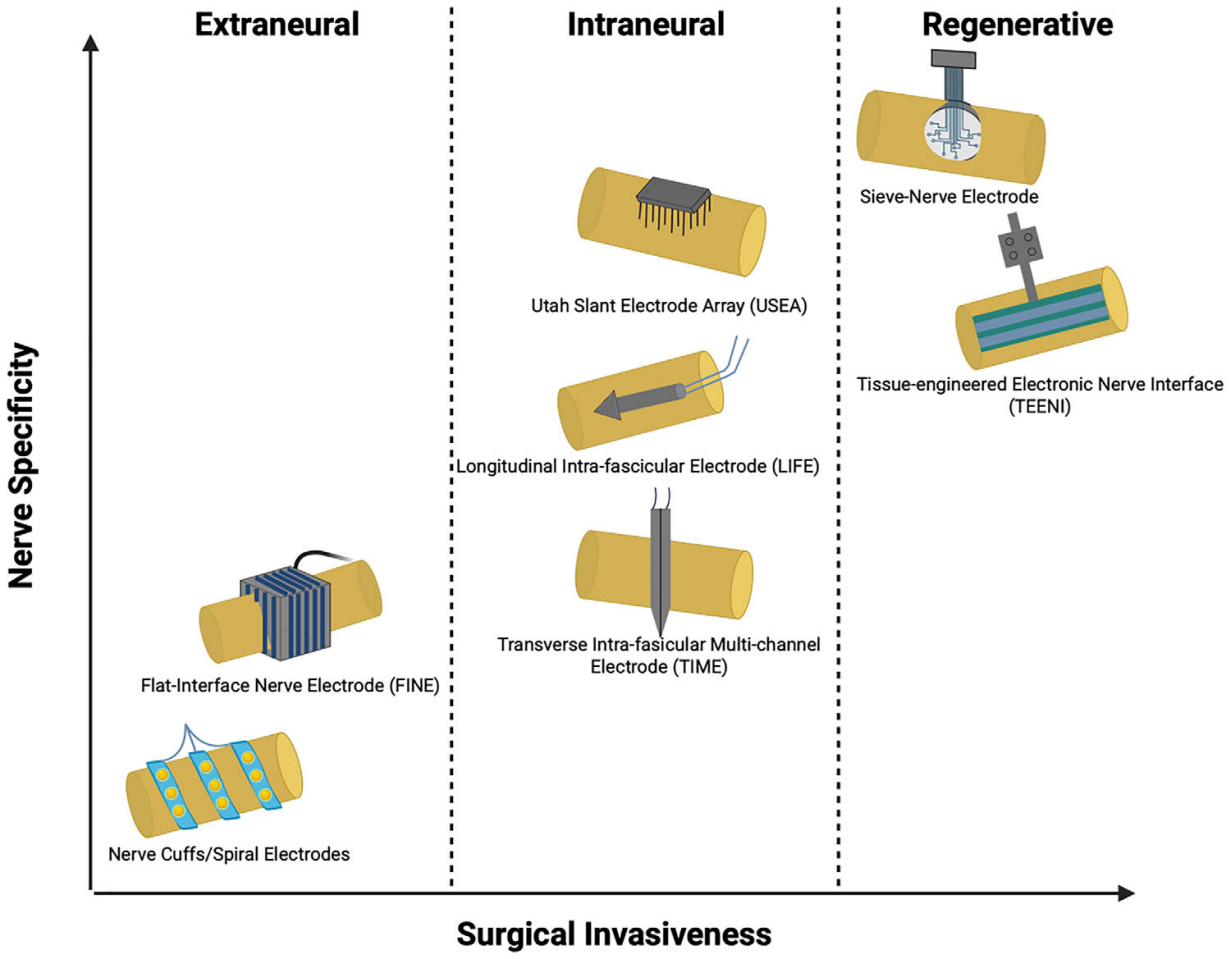
Classification of peripheral nerve interfaces by invasiveness and selectivity. The diagram shows neural interface categories: extraneural (FINE, Nerve Cuffs), intraneural (USEA, LIFE, TIME), and regenerative (Sieve, TEENI). Selectivity generally increases with invasiveness and risk of nerve damage. Extraneural devices wrap around or contact the nerve surface, intraneural devices penetrate or lie within fascicles, and regenerative interfaces require axonal regrowth through the device.

Nerve interfaces have existed since 1974, when Frank Clippinger implanted single‐channel cuff electrodes around the median and femoral nerves [94, 95]. Today, neural interfaces demonstrate stability for up to seven years and contain hundreds of channels [96]. Multielectrode arrays (MEAs) form the foundation of many interfaces, coated with natural (collagen, gelatine, chitosan) or synthetic films [97, 98, 99]. Cells may be attached to the devices by direct surface coating through methods such as photolithography and microcontact printing [97]. Device design has progressed in parallel, miniaturized power sources, implanted pulse generators, and battery‐free wireless telemetry using radio‐frequency power and infrared data, improve longevity while reducing size [100]. Another innovation, osseointegration, places sensorimotor interfaces directly into bone, as in the e‐OPRA device, improving precision and reducing reliance on surface electrodes, though users so far still depend on visual feedback, skewing outcomes [101, 102]. Together these advances have enabled remarkable restoration of sensory capabilities, including multiple USEAs restored 5 degrees of freedom and up to 131 proprioceptive and cutaneous sensations [103].

The quality of recording and stimulation depends on how the electrode's material properties match the nerve, and these properties can be tied directly to sensory function. Charge injection capacity sets the maximum safe stimulation amplitude, and therefore how selectively afferents can be recruited, materials such as PEDOT with parylene‐C raise injectable charge at small active sites for better selectivity [104]. In one application, PEDOT, whose high charge capacity and low impedance come from oxygen/sulfur doping, bridged the electrical conduction of the electrode and the ionic conduction of the nerve to enable an RPNI for closed‐loop control [104]. Low impedance improves recording fidelity and signal‐to‐noise ratio. Surface topography and roughness, often increased by electrodeposition, enlarge effective contact area for closer tissue contact [105]. Above all, Young's modulus mismatch governs chronic performance. Rigid materials such as silicon (∼130–190 GPa) or polyimide and parylene‐C substrates (∼1–10 GPa) are orders of magnitude stiffer than nerve tissue (∼200–400 Pa) shown in Table 2. Notably, polyimide is used for thin‐film electrodes, but is not approved for chronic implantation, posing a regulatory challenge for translation [105]. Table 2 benchmarks the representative materials and platforms, and Figure 7 summarizes how these properties propagate into integration and sensory outcomes.

**TABLE 2:** Benchmarking of representative neural‐interface materials.

Categories	Materials	Young's modulus / mechanical match	Charge injection capacity (CIC)	Impedance at 1 kHz	Chronic stability	FBR	Clinical maturity	Refs.
Structural substrates & encapsulants	Silicone elastomers/medical‐grade silicone, e.g., PDMS	1–50 MPa	N/A	N/A	Excellent	Low to moderate	Very high	[159, 160, 161, 162, 163]
	Polyimide (PI)	1–10 GPa	N/A	N/A	Moderate to good	Moderate	Moderate to high	[159, 160, 161, 162, 163, 164, 165, 166]
	Parylene‐C	1–4 GPa	N/A	N/A	Moderate	Moderate	Moderate	[160, 161, 162, 163, 164, 165, 167]
	Liquid crystal polymer (LCP)	2–10 GPa	N/A	N/A	Good	Low to moderate	Low to moderate	[162, 168, 169]
	Rigid silicon (Si)	130–190 GPa	N/A	N/A	Moderate	High	Moderate to high	[160, 161, 163, 164, 165, 170, 171, 172]
Inorganic conductive elements	Platinum/platinum‐iridium (Pt/PtIr)	170–230 GPa	Low to moderate; ∼0.05–0.15 mC/cm^2^	Moderate to high; ∼50 kΩ–1 MΩ, geometry‐dependent	Excellent	Moderate	Very high	[168, 173, 174, 175, 176, 177]
	Iridium oxide (IrOx; AIROF/SIROF)	∼300 GPa	High; ∼1–9 mC/cm^2^	Low to moderate; ∼5–100 kΩ, geometry‐ and coating‐dependent	Good	Moderate	Moderate to high	[159, 168, 174, 175, 178]
	Titanium nitride (TiN)	∼260–450 GPa	Moderate to high; ∼0.5–4.45 mC/cm^2^	Low to moderate; geometry‐ and coating‐dependent	Good to excellent	Moderate	Moderate to high	[159, 174, 178]
	Gold (Au)	∼79 GPa	Low; ∼<0.067–0.3 mC/cm^2^	Moderate to high; can be low when roughened/electroplated/nanoporous; geometry‐ and surface‐dependent	Moderate	Moderate	High	[159, 176, 179]
	Tungsten (W)	∼260–460 GPa	N/A (Unsuitable for stimulation)	High; ∼0.3–5 MΩ, geometry‐dependent	Moderate	High	Moderate	[176, 180]
	Stainless steel	∼190–205 GPa	Low; ∼0.02–0.05 mC/cm^2^	Moderate to high; ∼50 kΩ–1 MΩ, geometry‐ and surface‐dependent	Moderate to poor	Moderate to high	Moderate to high	[105, 174, 179, 181]
Soft, organic, and composite conductors	PEDOT	∼1 MPa–2 GPa	High; ∼1.0–15.0 mC/cm^2^	Low; often tens of kΩ for microelectrodes, geometry‐ and coating‐dependent	Poor to moderate	Low to moderate	Moderate	[166, 174, 177, 182, 183, 184]
	Polypyrrole (PPy)	∼0.1–1 GPa	High; ∼1–7.5 mC/cm^2^	Low to moderate; ∼7–10 kΩ, geometry‐ and formulation‐dependent	Poor to moderate	Low to moderate	Low to moderate	[177, 185]
	Carbon nanotubes (CNTs) / Graphene‐based conductive composites	∼1 TPa	High; ∼1–4 mC/cm^2^	Low to moderate; often tens to hundreds of kΩ, geometry‐, porosity‐, and coating‐dependent	Moderate	Low to moderate	Low to moderate	[160, 177, 186]
	Conductive Hydrogels	tunable from kPa to MPa	High; ∼1–9 mC/cm^2^	Low to moderate; can reach ∼1–50 kΩ, geometry‐ and hydration‐dependent	Poor to moderate	Low to moderate	Low	[159, 160, 168, 177, 182, 183]
Bioactive, anti‐inflammatory, and tissue‐integrative materials	ECM‐mimetic coatings (e.g., laminin, fibronectin, RGD)	N/A	N/A	N/A or indirect	Poor to moderate	Low to moderate	Low	[177]
	Natural Biopolymers (e.g., collagen, chitosan)	∼2–120 kPa	N/A	N/A or indirect	Poor to moderate	Low to moderate	Low to moderate	[168, 177, 187]
	PEG / zwitterionic antifouling coatings	N/A	N/A	N/A or indirect	Poor to moderate	Low	Low to moderate	[177]
	Dexamethasone‐eluting polymers	N/A	N/A	Indirect	Moderate	Low	Low to moderate	[184]
	PLGA/PCL/silk fibroin	Hundreds of MPa to GPa	N/A	N/A or indirect	Moderate	Low to moderate	Low to moderate	[160, 177]

*Nerve tissue*: 200–400 Pa [160]. *N/A*: The material is not typically used as the exposed charge‐injecting electrode surface or mechanically dominant component for that metric. *Impedance and CIC*: Only reported when the material directly forms the conductive electrode‐tissue interface; otherwise, effects are described as indirect or device‐dependent.

**FIGURE 7:**
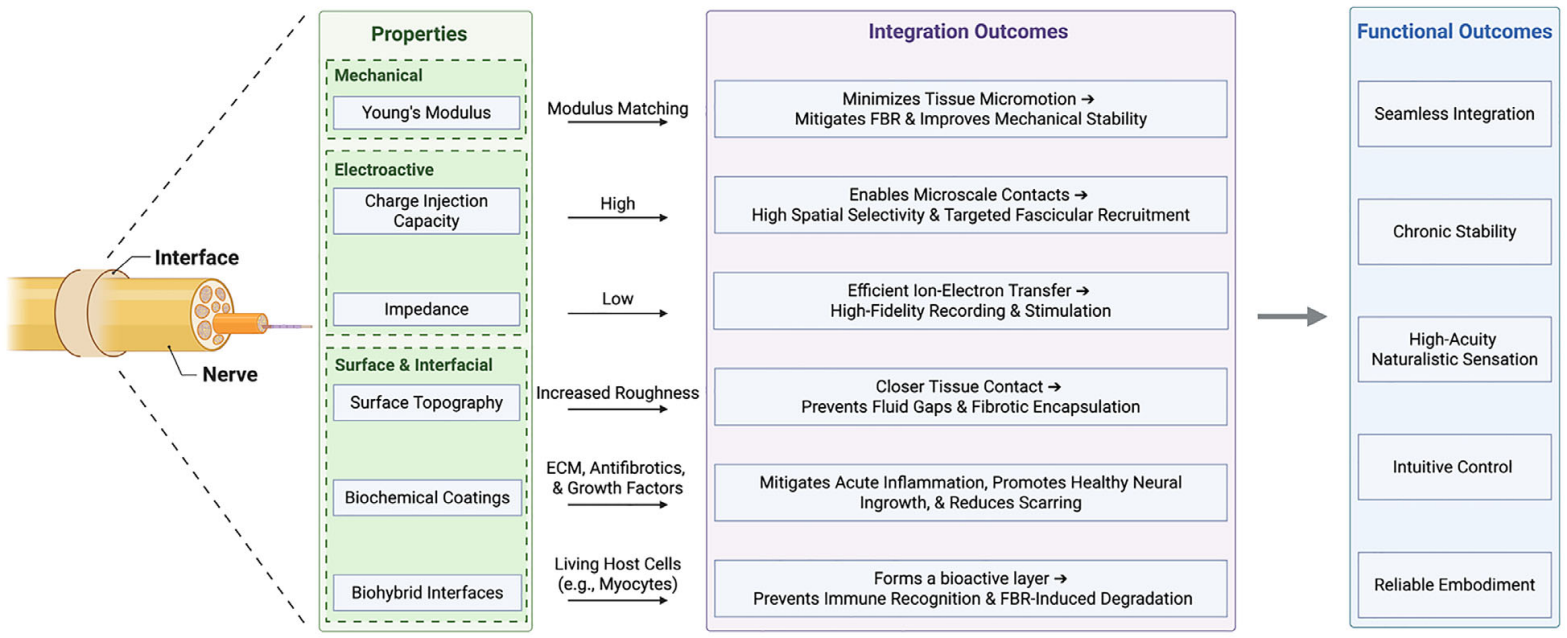
Conceptual framework of how interface properties shape sensory outcomes. Material and design properties, grouped into mechanical, electroactive, and surface/interfacial, propagate through their integration consequences, including mitigated FBR, mechanical stability, spatial selectivity, and high‐fidelity signal transfer. These integration outcomes in turn determine the functional outcomes of seamless integration, chronic stability, naturalistic sensation, intuitive control, and embodiment. The schematic illustrates how the engineering of the device‐nerve inteface ultimately shapes restored sensory perception.

#### 5.2.1 Material‐Based Strategies for Mitigating Foreign Body Response

FBR is the single most important factor in long‐term, chronic implant performance. Fibrotic encapsulation not only increases electrode impedance, but physically dislodges the electrode from its target, requiring repeated recalibration, burdening patients in time and money. Preventing and reducing chronic fibrosis therefore begins at the material interface itself. Much of the effort has gone into reducing the stiffness mismatch between the device and nerve, in turn limiting the micromotion that promotes inflammation, by incorporating soft conductive polymers, hydrogels, and ultra‐thin flexible substrates [106]. Surface chemistry has also been altered to drive integration of implants rather than encapsulation through extracellular‐matrix and anti‐fibrotic coatings, together with adhesion proteins and growth factors, such as laminin, collagen, NGF, and BDNF. Others have tried releasing anti‐inflammatory or anti‐fibrotic drugs locally, suppressing the early inflammatory cascade before it turns into fibrosis [106]. Throughout, any chronic implant, must remain nontoxic and non‐immunogenic, degrading only in a controlled manner and releasing no reactive byproducts. The more recent development of biohybrid interfaces pushes these strategies one step further, instead of coating or softening a foreign surface, it gives the body something it recognizes by seeding living cells onto the implant to form a biologically active layer, creating real integration.

#### 5.2.2 From Implant Challenges to Biohybrids

Implanted interfaces lose effectiveness over time due to electrode movement, impedance shifts, FBR, disease progression, and neuroplastic change, and current stimulation lacks the asynchronous, stochastic character of natural firing [107]. Biohybrids, interfaces that combine living cells with electrodes, address this by seeding cells onto the implant to form a biologically active layer that reduces FBR‐driven degradation, as shown in Figure 8. This principle has taken several forms. Regenerative sieve‐type electrodes, placed at a nerve stump, guide axons through holes for recording and stimulation, and may be loaded with growth factors for direction [108]. Embedding such implants in remodelable collagen matrices provides a 3D scaffold for regeneration and seamless integration [47]. Similarly, RPNIs have integrated autologous myoblasts within a scaffold to create a stable interface that prevents neuroma formation while amplifying neural signals [109]. More recent work records reinnervating axons through cultured myocytes integrated directly onto the device, forming an engineered biohybrid layer [110]. For any of these to succeed chronically, the cells and the electronics must remain stable in the physiological environment without degrading or provoking an immune response [47]. Since most biohybrid work to date targets motor restoration, sensory biohybrids and closed‐loop systems with real‐time feedback remain an open and promising direction.

**FIGURE 8:**
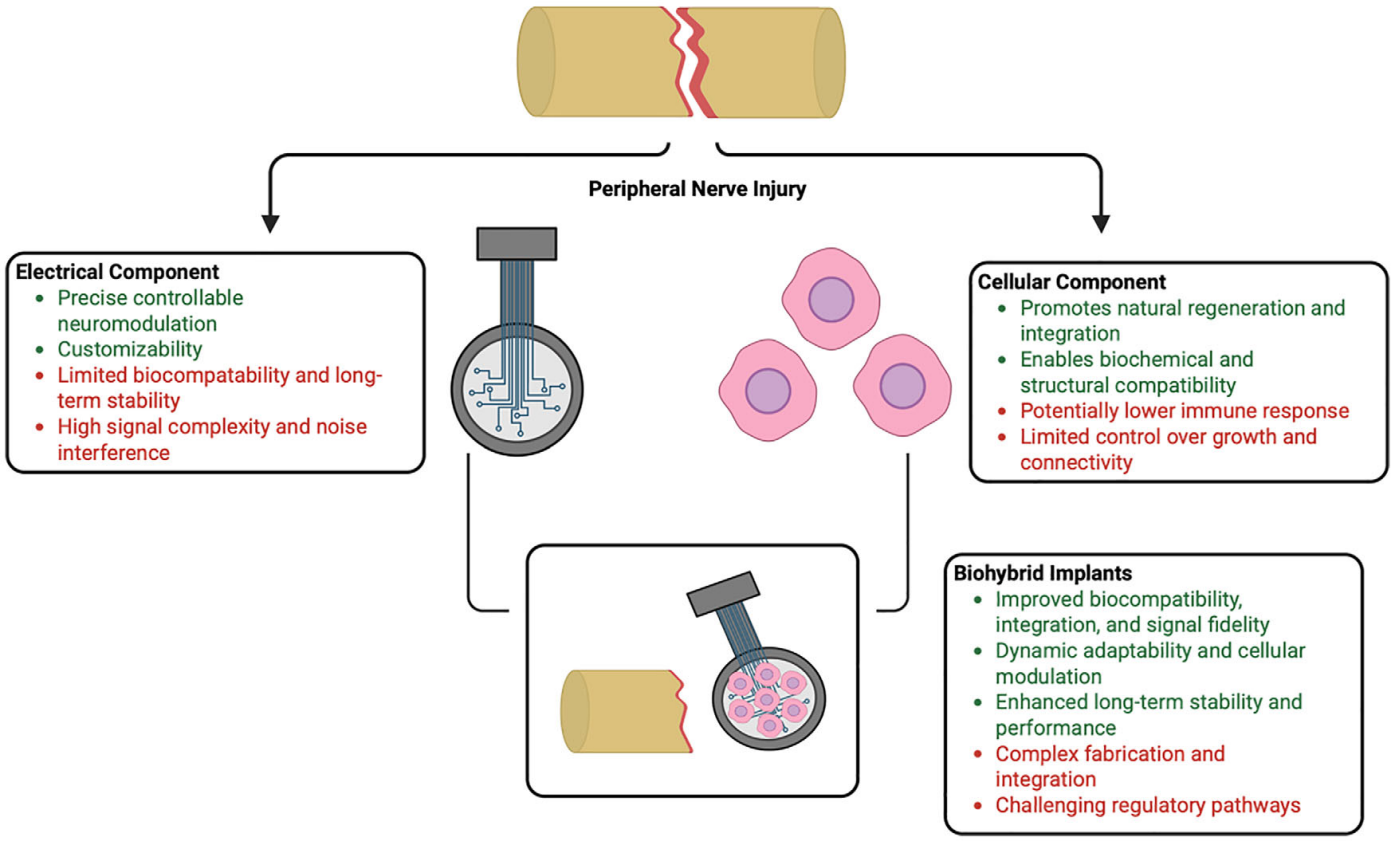
Biohybrid implants combine neural interfaces with cell transplantation to restore neurological function. Implantable devices enable stimulation and recording but have limited spatial reach. Transplanted cells can integrate into host networks but lack controllability. Biohybrid systems merge these strengths, offering integration and external modulation, though they pose significant design challenges.

## 6 Integration and Embodiment

### 6.1 Embodiment

Embodiment is the psychological integration of a prosthetic limb into one's body schema, a preconscious internal image of one's body [111]. It involves alignment between what is seen and felt, a sense of ownership, and agency over the limb [8]. When embodiment occurs, the prosthesis is perceived as part of the body that they have control over, an extension instead of a tool, involving neural changes that incorporate the device into sensorimotor processing [8]. This allows tasks to feel more intuitive and helps amputees feel whole again [8]. However, not all users experience embodiment, even with advanced prosthetics. Sensory feedback, especially congruence between visual and tactile feedback, improves embodiment, while mismatches can make the prosthesis feel foreign and impair use [112]. Studies using supernumerary robotic digits show that even nonanatomical devices can be embodied when sensorimotor cues align [113]. Proprioceptive feedback is essential for embodiment, even with vision and more so when task complexity increases [114].

To enhance embodiment, techniques such as mirror therapy, robotic rehabilitation, and immersive virtual reality (VR) are used [115, 116]. Mirror therapy uses the reflection of the intact limb to create the illusion that the phantom limb is moving [115]. VR offers controlled visual and tactile stimulation, spatiotemporal resolution control, repeatability of experiments, and effective training [116]. However, even slight visual mismatches in VR can disrupt embodiment [117]. These methods aim to better align sensory signals and promote ownership and control of the prosthesis.

### 6.2 The Rubber Hand Illusion

The rubber hand illusion (RHI) has helped reveal how limb ownership and sensory integration work [60, 118]. Synchronized stroking of a visible rubber hand and a hidden real hand creates the illusion that the rubber hand is part of the body. Previously, it was unknown whether the tactile sensation was felt in the rubber or real hand. One study showed the sensation referred to both simultaneously [118]. Interestingly, upper limb amputees can experience this illusion through synchronous stimulation of the stump and prosthesis, despite the classic RHI requiring identical stimulation points [60]. This may result from postamputation plasticity and less precise somatotopic organization, causing cortical zones for nearby body parts to overlap [119]. The illusion weakens if the rubber hand is not aligned or if the tactile stimulus differs in direction [120].

Self‐touch can enhance the illusion and promote embodiment [121]. Affective touch, vital in social interaction, also increases embodiment and supports realistic sensory restoration [122]. Ideally, prosthetic fingertips would include tactile sensors to provide everyday sensations and continuously reproduce the illusion.

The RHI highlights the brain's capacity to integrate multisensory inputs to create embodiment through synchronous and congruent visual and tactile feedback [123]. The embodiment can be so strong that injuring the rubber hand can trigger a fear reflex in amputees, as if their hand was injured [124]. Its strength varies with time since amputation and phantom limb sensations [60].

Recent advancements include visuomotor feedback and active movement to enhance the RHI [125]. Synchronizing movement between real and fake limbs, rather than just applying tactile stimuli, can strengthen ownership, showing that at least two sensory modalities are needed to induce embodiment [125]. Yet, some amputees experience phantom limb touch from only the visual stimulus approaching the hand without being touched, raising questions about whether multisensory input is always necessary [126]. Similarly, active movement of the rubber hand can amplify the RHI [125].

It is important to note that much about RHI and embodiment is still unknown. For instance, in the original study, embodiment faded quickly after tactile stimulation stopped, despite continued visual attention [125]. In contrast, real‐life embodiment does not require conscious attention lessening the cognitive load, which would make prosthetic use more seamless [127]. Furthermore, caution must be taken when interpreting RHI results, as the illusion is temporary, the rubber hand is immobile, and the user doesn't wear it [8]. Additionally, data from the experiment is often subjective, relying on qualitative user reports that may conflict with objective measures like temperature, reaction time, or cortical activity [8, 128, 129]. Better standardized metrics are needed to improve study reproducibility and allow comparisons across different experimental paradigms.

### 6.3 Proprioceptive and Tactile Feedback for Embodiment

Proprioceptive and tactile feedback are critical for motor control, embodiment, and psychological well‐being. Synchronous mechanotactile feedback enhances ownership and location awareness, while proprioception increases agency [130]. Sensory feedback also reduces perceived prosthesis weight, improving comfort, usability, and embodiment. In upper limbs, it enhances grasp control, while in the lower, it reduces falls, improves walking speed, and stabilizes gait [127].

Traditional approaches include sensory substitution, intraneural stimulation, and somatotopic tactile feedback. Sensory substitution electrically evokes cutaneous sensations to convey limb posture, offering an alternative to direct neural stimulation, which often causes paraesthesias [131]. Intraneural stimulation, by selectively activating afferent fibers, reduces cognitive load and fall risk, outperforming noninvasive methods [68, 127, 130, 131]. Nonetheless, challenges remain in eliciting natural, selective stimulation without causing unwanted muscle contractions and generating enough highly selective channels to convey the information of 17,000 cutaneous afferents in the hand [132]. To address this, recent work focuses on biomimetic sensory encoding, which better mimics natural tactile signals and improves feedback reliability and usability in prosthetics [107].

### 6.4 Neuromorphic Devices and Machine Learning

Neuromorphic technologies mimic the brain's spatiotemporal processing using event‐based, parallel, and in‐memory computing, such as encoding touch as spiking neurons [133, 134]. These devices use CMOS along with magnetometric, stretchable, and organic electronics to form neuronal and synaptic‐like circuits, reducing power by co‐distributing memory with processing [135, 136]. The co‐distributing avoids the inefficiency of biomimetic approaches reliant on bulky, expensive digital processors [134]. While replicating natural neural firing remains difficult, spatial and temporal aspects of activation modulated by stimulation parameters are feasible [137].

Biomimetic stimulation patterns aim to produce more natural feedback and intuitive sensations. One group developed a CMOS‐based neuromorphic device for real‐time, energy‐efficient encoding of physical stimuli in a compact nature [138]. Another developed a chronic, fully implantable multichannel stimulator using CMOS and battery‐free telemetry [139]. Today well‐known neuromorphic devices include Neurogrid, Loihi, and BrainScales2 illustrating the rapid progress in large‐scale neural stimulation [140, 141, 142]. Future neuromorphic efforts target restoring neuronal asynchrony, plasticity, and neuromuscular reflexes [135, 143, 144], and integration with treatments like noninvasive TENS [145].

Machine learning complements these hardware advances by addressing the calibration and adaptation problems that fixed stimulation cannot. Current calibration approaches are trial‐and‐error, requiring frequent clinical adjustments, highlighting the need for subject‐specific machine learning algorithms for calibration [146]. Techniques such as Bayesian optimization and reinforcement learning enable real‐time adjustments that reduce the need for manual recalibration [147]. Machine learning is already being integrated into systems like TENS [147]. More advanced algorithms have also shown to improve metabolic consumption, confidence, and gait stability, while reducing cognitive load and setup time [56, 147]. More broadly, machine learning is being used to refine neural encoding models and translate artificial sensor data into biomimetic stimulation, complementing the goal of neuromorphic devices to generate naturalistic signals in real time [146, 148].

Despite these advancements, reproducing natural firing exactly may not be achievable and the limits of machine learning models are becoming clear. Real‐time closed‐loop recalibration is demanding and must run within strict latency and power budgets on an implant. Patient‐to‐patient variability limits how well models transfer between users, and is compounded by scarce, costly labeled neural‐interface data, and limited interpretability, knowing why a model selected a given stimulation pattern, complicates safety and regulatory approval once a paradigm must adapt online [149, 150]. Machine learning and neuromorphic encoding are therefore best understood, for now, as simply tools to reduce trial‐and‐error calibration and approximate naturalistic feedback, not as substitutes for selective, stable, biocompatible hardware.

## 7 The Special Difficulty of Proprioceptive Restoration

Much of the field has concentrated its effort on tactile restoration, but the harder and arguably more important frontier is proprioception for several reasons. It is, for one, the least measurable sensation, and therefore the least understood. Tactile afferents, when stimulated, produce consciously reportable percepts that scale with firing rate, but proprioceptive afferents evoke no discrete, reportable sensation at all [151]. The objective, trial‐and‐error tuning used to develop tactile encoding strategies cannot be applied here. Researchers must instead rely on subjective reports that vary greatly from person to person [152]. Even setting measurement aside, the signal itself is intrinsically dynamic and closed‐loop, continuously encoding the change in position, velocity, and force, which demands real‐time bidirectional encoding rather than only fed discrete events [14]. Substitution itself doesn't help, unlike in tactile substitution that works because residual or alternative skin can host a sensory map, proprioception has no equivalent surface, meaning that substitutes such as skin‐stretch at their best are indirect [63]. A successful encoding strategy would still at the end of the day face the anatomy, as muscle spindles and GTOs lie within muscle and tendon rather than at an accessible nerve surface, they make it difficult to engage them selectively without also activating motor fibers [91].

There is, however, real promise in AMI because it preserves the native spindle‐GTO pathways, generating physiologically meaningful position, speed, and force signals, restoration that TMR and RPNIs cannot provide [34]. Where a more direct neural route is required, DRG stimulation, have recruited proprioceptive afferents experimentally and been already FDA‐approved for chronic pain allowing adoption with minimal regulatory hurdles [14, 23, 24, 153]. When surgery is not an option, noninvasive skin‐stretch devices remain the leading substitution [63]. Ultimately, progress will depend on developing objective, closed‐loop metrics of proprioceptive accuracy to replace the subjective reporting, alongside continued advances in closed‐loop technology [8, 128, 129].

## 8 Conclusions

Several design principles emerge from this review for where the field should be heading. Wherever the residual architecture allows, feedback should be matched in both modality and location to the function that was lost, because homologous, somatotopic signals minimize training and maximize the restoration of natural perception [26, 60]. At the physical interface, device‐nerve interface stiffness as a crucial design principle, favoring soft conductive polymers, hydrogels, flexible substrates, and anti‐fibrotic or ECM coatings to limit FBR and preserve chronic stability, while using high‐charge injection materials to selectively recruit afferents [106]. Proprioception should also be explicitly included from the beginning instead of treated as an afterthought to touch, since restoring touch means little without proprioception complementing it. Strategies should be built around preserving the natural pathways, such as AMI, and contain a new set of standardized, objective, closed‐loop metrics [34]. On the signal side, interfaces should reproduce the spatiotemporal structure of natural firing and conduct closed‐loop real‐time feedback [107]. Machine learning is best used to reduce trial‐and‐error calibration and compensate for impedance drift, not to substitute for selective, stable implants [146].

Finally, translation should be a goal kept in mind from the start because the barriers to getting there are real. Scalability is limited by material costs, fabrication complexity, and regulatory barriers. Powering implants remains difficult due to limited battery lifespans, though emerging solutions show promise, including glucose fuel cells, piezoelectric generators, and inductive coupling for wireless energy transfer [154, 155, 156]. The FDA also classifies implantables as Class III devices, unlike traditional Class I prostheses, requiring extensive testing, increasing costs and timelines. High abandonment rates, extensive training needs, and limited usability hinder adoption too, which is why user needs, intuitive designs, and minimal calibration should be prioritized from the beginning rather than retrofitted later. On the study design side, standardized metrics, larger sample sizes, and direct comparisons across methods are essential. Underlying it all, PNIs range from partial to complete transections, so no single approach will address every injury type optimally.

Beyond these practical and regulatory barriers, adoption also raises ethical concerns, including high cost, accessibility, and questions around agency, autonomy, identity, and privacy [157]. There are concerns surrounding long‐term neural plasticity and unwanted psychological consequences of neural interfaces, especially those implanted. These implanted devices require clear patient education on the risks like neuroinflammation, device failure, and privacy breaches. Researchers must develop regulatory frameworks to address the ethical challenges and ensure equitable access to these emerging technologies [157]. Ultimately, the future of peripheral nerve restoration lies in patient‐centered interfaces that let individuals not only move but feel again.

## Author Contributions

This project was conceived and designed by D.G.B. and S.S. S.S. and S.E.H. prepared the project and developed the search strategy. S.S. screened studies and determined inclusion. S.S., S.E.H., and K.L. drafted the manuscript. D.G.B. and G.G.M. edited the manuscript and provided oversight for the project.

## Figure Source Statement

All figures were created using BioRender.com under a paid license.

## Consent

All authors have read and approved the manuscript and consent to its publication.

## Conflicts of Interest

The authors declare no conflicts of interest.

## Data Availability

This manuscript is a review of existing literature in the field through the online databases of Embase and MEDLINE. No new data were created or analyzed in the preparation of this article.
